# Language outcome related to brain structures in school-aged preterm children: A systematic review

**DOI:** 10.1371/journal.pone.0196607

**Published:** 2018-06-04

**Authors:** Lottie W. Stipdonk, Marie-Christine J. P. Franken, Jeroen Dudink

**Affiliations:** 1 Department of Otorhinolaryngology at Erasmus Medical University Centre-Sophia Children’s Hospital, Rotterdam, Netherlands; 2 Division of Neonatology, Department of Pediatrics at Erasmus Medical University Centre-Sophia Children’s Hospital, Rotterdam, Netherlands; 3 Division of Neonatology, Department of Pediatrics at UMCU-Wilhelmina Children’s Hospital, Utrecht, Netherlands; Cincinnati Children's Hospital Medical Center, UNITED STATES

## Abstract

Preterm children often have language problems. This atypical language development is probably due to atypical brain development. We conducted a systematic review to provide an overview of the extensive and diverse scientific literature on the relations between language outcome and underlying brain structures in school-aged preterm-born children. Embase, Medline Ovid, Web of Science, Cochrane central and Google scholar were searched for relevant studies. Inclusion criteria were: cases are school-aged preterm children; structural MRI (T1- and T2-weighted sequences) or DTI used in combination with a neurocognitive language test; publication in an English-language peer-reviewed journal. Correlational measures between language scores and brain volume or fractional anisotropy of a brain structure were extracted. 23 studies were included. The relations between oral language, verbal fluency and/or written language and MRI/DTI measurements of white matter, gray matter, cerebellum, corpus callosum and/or the fasciculi are presented. Oral language skills and verbal fluency appear to be related to the corpus callosum. Oral language skills are also related to the uncinate fasciculus. There seems to be no clear relation between cerebellar development and verbal fluency skills. Not one single brain area is responsible for atypical language development, but several brain areas and their connections are essential. For future research it is recommended to relate brain areas to oral language skills on a microstructural level in preterm children. We also recommend to use language tests in which it is possible to distinguish between several language domains, such as perceptive and expressive language.

## Introduction

Technological advances and combined efforts of obstetricians and neonatologists have resulted in improved survival for preterm infants [1]. Nowadays, very preterm children (<32 weeks) represent 1%-2% of all live births in developed countries [2]. These children are at risk for neurocognitive deficits even later in life. Depending on gestational age and neonatal complications, up to 30% of very preterm survivors in developed countries will experience significant long-term neurodevelopmental problems, such as cognitive, motor or hearing impairment [3]. Subtler neurodevelopmental impairments, such as language disorders, learning disabilities, attention deficits, behavioral problems and social-emotional difficulties, occur even more often [3–9]. Almost 20% of very preterm children are diagnosed with language disability at school age (6 to 17 years) and more than 50% require additional education [10]. Two recent meta-analyses showed that problems with complex language functions, such as storytelling, even increase at ages 3 to 13 years [6, 11]. These outcomes are alarming since language development is extremely important for academic achievements and communication in everyday life.

The atypical language development in preterm children is most likely a consequence of atypical brain development [10, 12]. Several magnetic resonance imaging (MRI) studies showed macrostructural (e.g. measurements from T1- and T2 weighted structural MRI sequences) and microstructural (e.g. diffusion weighted MRI sequences) deviations in brain development in preterm children in childhood and adolescence [12]. As compared to term-born controls, very preterm children had significantly smaller total brain volume, white matter volume, gray matter volume, cerebellum, hippocampus and corpus callosum. Furthermore, preterm birth is associated with a reduction in cortical folding. In a recently published systematic review of the association between very low birth weight (VLBW) and brain structures and cognitive function impairments, the authors concluded that both brain structures and cognitive functions are more often atypically developed in VLBW children [13]. However, they did not look for association measurements between these two parameters and they did not include language outcome. Therefore, the association between atypical brain development and language skills remains unclear. Recently Kwon et al. reviewed literature about association measures between functional connectivity and language disorders [10]. The authors suggest that there are alterations in the functional organization of language in preterm children and that these alterations in the developing brain are both proximate and long lasting. However, to our knowledge no systematic review has been published about association measures between structural MRI measures and language development in preterm children, whilst many studies have addressed relations between different MRI brain structures and several language domains. These MRI studies are diverse, however, since they focus on the relation between two measurements (language outcomes and brain structure measurements), which both can vary. A clear overview of all these results could be of great use to clinicians and researchers in the field. It can contribute to a better understanding of the actual relations between language and the brain in preterm children and set directions for consistent and high-quality research. Hence, the aim of the systematic review presented here is to provide an overview of what is currently known about language outcome of school-aged preterm children and the associations with their brain structures measured on MRI.

## Method

### Selection of studies

**Fig 1:**
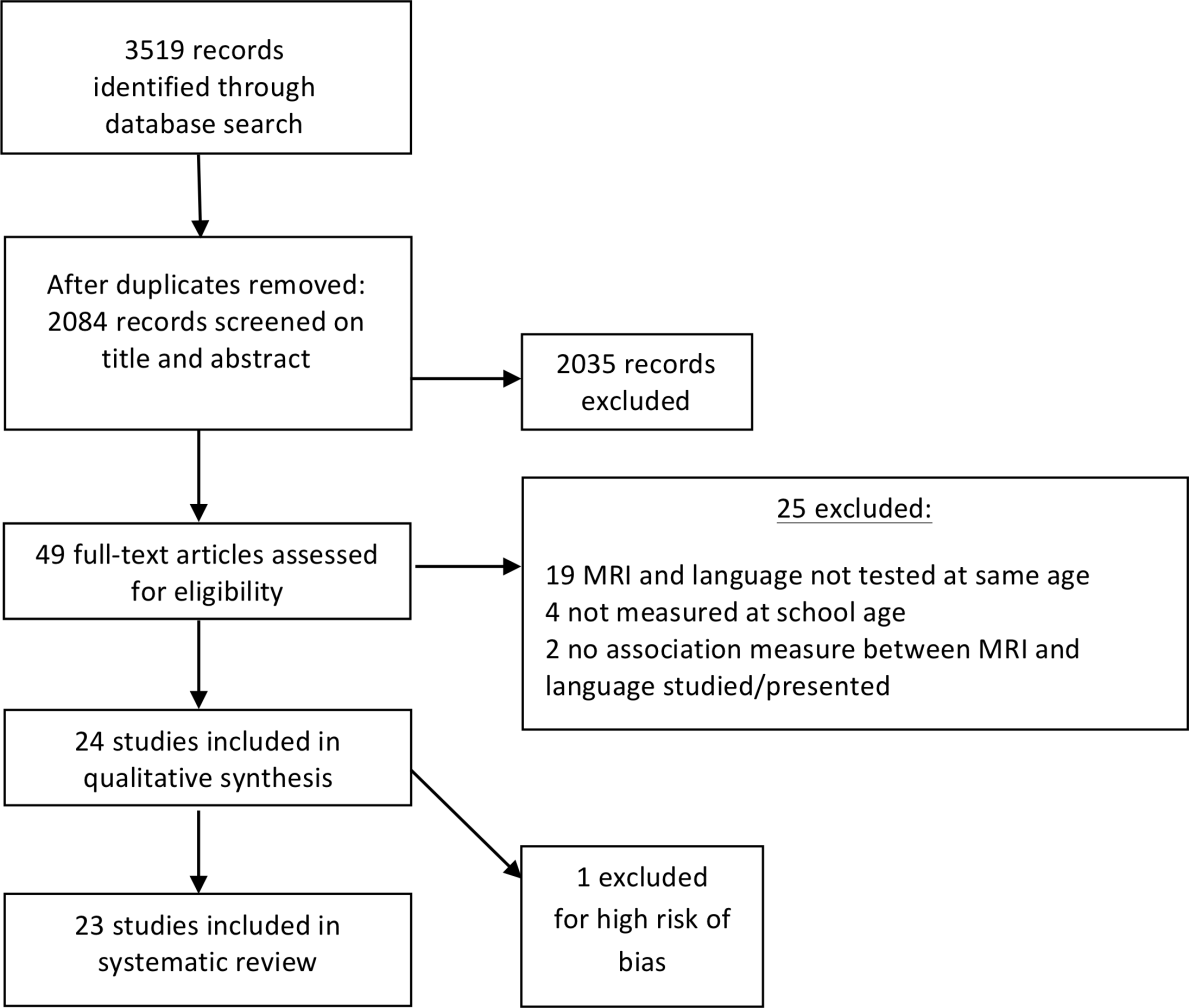
Flow diagram of study selection.

The computerized Embase, Medline Ovid, Web of Science, Cochrane central and Google scholar databases were searched for articles in January 2017 (and again in September 2017 to detect recently published articles) combining the search terms *neurological*, *neurophysiology*, *neurobiology*, *forebrain*, *brain* AND *speech*, *language*, *verbal*, *linguistic*, *reading*, *writing*, *literacy*, *illiteracy*, *vocabulary*, *grammar*, *phonology*, *dyslexia* AND *premature*, *prematurity*, *preterm*, *"low birth*, *birthweight"*, "small*gestational age". In Fig 1 the flow diagram of the study selection is presented. The search yielded 2083 unique articles. In S1 Text the complete search protocol can be found for all databases. Based on screening of titles and abstracts, 2035 articles were excluded. 49 articles remained and were assessed for eligibility based on the following exclusion criteria: (1) study cases are not school-aged children (6–17 years) born preterm (gestational age (GA) <37 weeks); (2) brain structures not measured with structural MRI (T1- and T2-weighted scans) and/or DTI; (3) language not assessed at the same age as the MRI scan was made; (4) no correlational measure is published between language and brain volume or fractional anisotropy of a brain structure; (5) not published in an English language, peer-reviewed journal; (6) no sufficient study quality according to the Newcastle-Ottawa Quality Assessment Scale for cohort studies. A total of 25 studies had to be excluded based on these criteria, which resulted in 24 studies that were suitable for our data extraction and analysis (23 originally in January 2017, and 1 added in September 2017). Subsequently, one study was excluded since there was high risk of bias [14]; the population and main outcome measure of this study were overlapping with those of one of the other included studies [15]. Of these two studies, we included the most recently published one [15]. The main characteristics of the final 23 included studies are presented in Table 1.

**Table 1:** Study characteristics.

**Gäddlin et al.[16] 2008**	PT: 59FT: 57	<30 weeks	Down syndrome	15	T1w, T2w(6 different hospitals)	WM injury4-point scale	Oral language (WISC)Written language: reading	0
**Yliherva et al.[17] 2001**	PT: 41FT: 24	26–35 weeks	Rett syndrome Duchenne dystrophy	8	1,0 T1w	WM injury4-point scale	Oral language (ITPA, MT, TTC)	0
**Rushe et al.[18] 2001**	PT: 75FT: 21	<33 weeks	NA	14–15	GE 1,5 T1w, T2w	Injury3-point scale	Verbal Fluency (FAS, Object and Animal naming, BNT) Written language (SGRST)	0
**Skranes et al.[19] 1997**	PT: 18FT: 0	<1500 gram	Disabled children (such as CP)	6	Philips 1,5 T1w, T2w	Presence of periventricular gliosis, loss of white matter, ventricular dilatation and cortical atrophy	Oral language (WPSSI)	0
**Isaacs et al.[20] 2004**	PT: 65FT: 0	28,5 (1.2)	Neuromotor or neurosensory impairment	12–16	Siemens 1,5T2w	Brain volumes	Oral language (WISC)	WM: +par/temp,–fr*(VBM correlations*: *39*, *-69*, *28; p <* .*01)*GM:–par, +fr*(VBM correlations*: *±40*, *−70*, *30;* p*<0*.*01)*
**Nosarti et al.[21] 2008**	PT: 207FT: 104	<33 weeks	For controls: any history of neurological conditions including meningitis, head injury and cerebral infections	14–15	GE 1,5T1w	Brain volumes	Verbal Fluency (FAS, Object and Animal naming)	WM: +fr/tempGM:–fr/temp *(29% of variance*: *F = 2*,*3; p<0*.*0001)*
							Written language (SGRST)	WM: +fr/temp,GM:–fr/temp*(28% of variance F = 2*,*3; p<0*.*0001)*
**McCoy et al.[22] 2014**	PT: 26FT: 0	27,81 (2.0)	Inclusion: liberal transfusion group	13	Siemens 3,0T1w, T2w	Brain volumes	Verbal Fluency (COWAT)	WM females: +temp*(r*^*2*^ *Δ =* .*237; p <* .*05)*Cerebellum: 0
**Scott et al.[23] 2011**	PT: 218FT: 127	<33 weeks	NA	14–15	GE 1,5T1w, T2w	Brain volumes	Verbal Fluency (COWAT, Object and Animal Naming)	WM: 0fr
							Written language (SGRST)	WM: 0frGM: +fr*(z-score 4*.*98; p <* .*05)*
**Arhan et al.[24] 2017**	PT: 22FT: 24	28–33 weeks	Apgar score at 5 min >7; absence of major neonatal morbidity; absence of cerebral pathology such as IVH	9	Siemens 1,5T1w	Cerebellum and CC volume	Oral language (WISC)	Cerebellum: +*(Subtest comprehension*:*r =* .*93; p =* .*001)*CC: +*(subtest vocabulary*: *r =* .*91; p =* .*001)*
**Parker et al.[25] 2008**	PT: 65FT: 34	<33 weeks	IVH, drug exposure during pregnancy	15	GE 1,5T1w	Cerebellum volume	Oral language (WISC)	+*(r =* .*401; p =* .*008)*
							Verbal Fluency (COWAT, Object and Animal Naming)	0
**Narberhaus et al.[26] 2008**	PT: 52FT: 52	<33 weeks	IQ < 70, history of traumatic brain injury, CP or other neurological diagnosis, motor or sensory impairment that precluded neuropsychological assessment	10–19	GE 1,5T1w	CC volume	Oral language (WISC/WAIS subtest)	+*(for splenium*:*r =* .*32; p <* .*05)*
							Verbal fluency (COWAT)	+*(for genu*: *r =* .*37; p <* .*01) (for splenium*: *r = 0*.*32; p <* .*05) (for isthmus*: *r =* .*28; p <* .*05)*
**Nosarti et al.[27] 2004**	PT: 66FT: 51	<33 weeks	NA	14–15	GE 1,5T1w, T2w	CC volume	Oral language (WISC)	+*(for mid-posterior CC*: *β =* .*33; p =* .*009)*
							Verbal fluency (COWAT, Object and Animal Naming)	+*(total CC*:*β =* .*35; p =* .*006)*
							Written language (SGRST)	0
**Allin et al.[28] 20ˋ01**	PT: 67FT: 50	<33 weeks	NA	14–15	GE 1,5T1w	Cerebellum volume	Verbal Fluency (FAS, BNT)	0
							Written language (SGRST)	Reading: +*(β =* .*295; p =* .*019)*Spelling: 0
**Martinussen et al.[29] 2009**	PT: 50FT: 49SGA: 49	29,1 (2.7)	NA	15	Siemens 1,5IR	Brain volumes	Oral language (WISC)	0WM and Cerebellum: +SGA*(Stepwise regression*: *step 2*, *part r =* .*1066*, *F value = 6*.*53; p =* .*0142)*
**Brumbaugh et al.[30] 2016**	PT: 52FT: 74	34–36 weeks	Multiple birth, major medical disease, neurological injury, 5-minute Apgar score <7, neonatal sepsis, and birth weight <1500 g for late PT children and <2500 g for FT children	6–13	Siemens 3,0T1w	Brain volumes (WM, Cerebellum)	Oral language (WISC)	0
							Verbal Fluency (BNT, Object Naming)	0
							Written language: reading (WRAT)	0
**Caldú et al.[31] 2006**	PT: 25FT: 25	<33 weeks, 29.48 (2.52)	Mentally or physically disabled children	13	GE 1,5T1w	GM, WM and CC volume	Oral language (WISC/WAIS),	WM: 0GM: +*(r =* .*50; p <* .*05)*CC: 0
							Verbal fluency (RAVLT)	WM: 0GM: 0CC: 0
**Northam et al.[32] 2012**	PT: 50FT: 30	<33 weeks. 27(2)	NA	16	Siemens 1,5T1w, T2w	Brain volumes (CC, Fasciculi)	Oral language (PPVT, TROG)	CC: +*(Ancova F(2*.*72) = 20*.*5 p <* .*0001)*UF: +AF: 0
**Mullen et al.[33] 2011**	PT: 44FT: 41	28,3 (1.9)	IVH, PVL, low pressure ventriculomegaly, abnormal MRI findings, abnormal total ventricular CSF volume	16	Siemens 1,5	Brain volumes	Oral language (WISC, PPVT)	WM: 0AF: 0UF: +*(left*: *r =* .*314; p =* .*038*,*right*: *r =* .*336; p =* .*026)*
							Verbal Fluency (CTOPP)	AF: +*(left*: *r =* .*424; p =* .*004*, *right*: *r =* .*301; p =* .*047)*UF: 0
**Andrews et al.[34] 2010**	PT: 19FT: 9	24–36 weeks 30,5 (3,2)	NA	11	Siemens 3,0T1w	DTI	Written language : reading (WJTA)	Temp/par: +*(for passage comprehension*: *left*: *r =* .*417; p <* .*05*, *right*: *r =* .*459; p <* .*05)*CC: +*(for word identification*:*r =* .*553; p <* .*05for word attack*: *r =* .*537; p <* .*05)*
**Constable et al.[35] 2008**	PT: 29FT: 22	28,4 (2,0)	IVH, WM injury and/or ventriculomegaly	12	GE 1,5SPGR	DTI	Oral language (WISC, PPVT-R)	UF: +males*(for VIQ left*: *r = 0*.*513; p =* .*051*, *right*: *r =* .*635; p =* .*008for PPVT left*: *r =* .*511; p =* .*052*, *right*: *r =* .*619*, *p =* .*011)*UFright:–females*(for VIQ*: *r = -*.*744; p =* .*004for PPVT*: *r = -*.*759*, *p =* .*003)*
**Kontis et al.[36] 2009**	PT: 63FT: 45	<33 weeks	Left-handedness	15	GE 1,5	DTI	Oral language (WISC)	CC: 0
							Verbal Fluency (CVLT)	CC: +*(for CC body with intrusions item*: *r =* .*295; p =* .*029for Splenium with short delay*: *r =* .*312*, *p =* .*020for splenium with long delay*: *r =* .*273 p =* .*0*.*44for splenium with long delay free recall*: *r =* .*313*, *p =* .*020for splenium with intrusions*:*r = -*.*306*, *p =* .*023)*
**Skranes et al.[37] 2007**	PT: 34FT: 47	29,3 (2.7)	Trisomy 21	15	Siemens 1,5T1w	DTI	Oral language (WISC)	SLright: +*(r =* .*363; p<0*.*05)*
**Travis et al.[15] 2016**	PT: 26FT: 19	26,0–34,5	Active seizure disorder, hydrocephalus, receptive vocabulary score < 70, sensorineural hearing loss, and non-native speaker of English	9–17	GE 3,0T1w	DTI	Written language: reading (WJTA; BRSC)	AFleft: +*(for decoding*: *r =* .*606; p <* .*05)UFleft*: *+(for comprehension*: *r =* .*562; p <* .*05)*SL: +*(for decoding*: *right*: *r =* .*403; p <* .*05)*, *left*: *r =* .*466; p <* .*05for comprehension*: *left*: *r =* .*417 p <* .*05)*

In the correlation column, a ‘+’ refers to a positive correlation; a ‘-’ refers to a negative correlation; a ‘0’ refers to no significant correlation.

Abbreviations: AF = arcuate fasciculus; CC = Corpus Callosum; CSF = cerebrospinal fluid; f = females; fr = frontal lobe; FT = full-term; GE: General Electric; GM = gray matter; m = males; IVH = Intraventricular hemorrhage; NA = not applicable; L = left; par = parietal lobe; PT = preterm; PVL = periventricular leukomalacia; R = right; read = reading; SGA: small for gestational age; SL = superior longitudinal fasciculus; spel = spelling; spl = splenium; temp = temporal lobe; UF = uncinated fasciculus; WM = white matter.

### Quality assessment

Two authors (LWS and JD) independently assessed the methodological quality of the included studies according to the Newcastle-Ottawa Quality Assessment Scale for cohort studies (S1 Table). This scale assesses the quality of cohort studies from the selection of the population, the comparability of the study groups and the ascertainment of outcome of interest. The total rating score ranges from 1 to 9, with 9 being the most favorable. Any disagreement between the two assessors with regard to the total score was resolved by discussion. The quality scores of selected articles are presented in S1 Table. Overall quality was rated from 5 to 8 stars. To improve our reporting the PRISMA checklist was used (S2 Table).

### Outcome measures

#### Language outcome

Language is a very complex phenomenon which encompasses many different subdomains. Most language tests represent only one of these subdomains, assessed by associated language tasks. Therefore, not all language studies can be compared in a single, consistent way. Only studies that used the same language task, or comparable ones measuring the same language domain, can be validly compared. For example, composing and speaking a complex sentence is a task that is completely different from summing up words that start with an F, or spelling individual words–each of these three tasks requires skills from a specific language domain. Inevitably, the language tests used in the included studies vary widely. To be able to still validly compare study results we created three categories: oral language; verbal fluency and written language.

The oral language category includes tests that assess oral language ability, such as word and sentence comprehension and production, and vocabulary. Included tests are: Clinical Evaluation of Language Fundamentals-4 (CELF); Illinois Test of Psycholinguistic Abilities (ITPA); Morphological Test (MT); Peabody Picture Vocabulary Test (PPVT); Test for Reception of Grammar (TROG); Token Test for Children (TTC); Verbal scale of Wechsler Intelligence Scale for Children-III (WISC); verbal scale of Wechsler Adult Intelligence Scale (WAIS); verbal scale of Wechsler Preschool and Primary Scale of Intelligence (WPSSI).

The verbal fluency category includes tests that assess verbal (phonetic or semantic) fluency, which requires special use of executive functions in combination with language functions: Boston Naming Test (BNT); Controlled Oral Word Association Test (COWAT); Comprehensive Test of Phonological Processing (CTOPP); California Verbal Learning Test (CVLT); FAS-test; Object and Animal naming; Rey Auditory Verbal Learning Test (RAVLT); Stroop test TBAG version.

The written language category includes tests that target reading and spelling: Basic Reading Skills Cluster (BRSC); Schonnel Graded Reading and Spelling Test (SGRST); reading subtests of the Woodstock-Johnson III Test of Achievement (WJTA); reading score of Wide Range Achievement Test (WRAT).

#### MRI

We related language outcome in the above-mentioned three categories to the underlying brain structures. Different brain structures can be reliably measured *in-vivo* on structural (anatomical) T1- and T2-weighted MRI sequences–either manually, semi-automatically or automatically. Different software post processing tools are available for this purpose, allowing macro-structural measurements of brain structures. Diffusion tensor imaging (DTI) sequences allow visualization and quantification of white and gray matter microstructure. Several diffusion parameters can be derived from DTI results, but white matter integrity is most commonly estimated with fractional anisotropy (FA). FA is a scalar value between 0 and 1 describing the amount of diffusion asymmetry (anisotropy) within a voxel, defined in terms of its eigenvalues. FA = 0 means that diffusion is isotropic (i.e. it is unrestricted or equally restricted in all directions). FA = 1 means that diffusion occurs along one axis only and is fully restricted along all perpendicular directions.

### Data extraction

For each included study we extracted the published correlational measures between language scores and brain volume or fractional anisotropy of a brain structure. Most of the studies reported a Pearson’s correlation coefficient. A few studies also reported Spearman’s rho or stepwise logistic regression analyses as a correlation measure. The correlational measure had to correlate a language score (classified within one of the three categories discussed above) and a brain area that is addressed in at least two different studies. For example, total brain volume, brainstem volume and cerebral spinal fluid (CSF) were all studied only once [29–31], and therefore the respective findings are not presented in the cross table. Besides, these studies explicitly reported that they did not find any significant relations with language.

## Results

Four of the 23 included studies [16–19] addressed the relation between children’s language skills and white matter injury only, classified on either a 3- or 4-point scale. None of these studies found a significant association between this damage classification and language skills.

The remaining 19 studies used brain volume measurements or DTI to relate brain structures to language outcomes. A cross table (Table 2) shows the associations between language skills and brain structures reported in these 19 studies. A ‘+’ refers to a positive correlation, a ‘–’ to a negative correlation and a ‘0’ to no significant correlation.

**Table 2:** Study results.

				Arcuate	Uncinate	Superior Longitudinal
+SGA[29], +par/temp[20],–fr[20]	+[31], +fr[20]–par[20]	+spl[26], +[27]^,^ [24, 32]	+SGA[29], +[24, 25]		+[32, 33]L/R, +m[35],–f R[35]	+R[37]
0[29, 31]^,^ [30]^,^[33]		0[31, 36]	0[29]^,^[30]	0[32, 33]L/R		
+fr/temp[21] +temp_f[22]	–fr/temp[21]	+[26, 27, 36]		+[33]L/R		
0[30, 31], 0fr[23]	0[31]	0[31]	0[22, 25, 28, 30]		0[33]L/R	
+fr/temp[21] +temp/par[34]	–fr/temp[21]+fr[23]	+[34]	+read[28]	+L[15]	+L[15]	+L/R[15]
0[30], 0fr[23]		0[27]	0[30], 0spel[28]			

A ‘+’ refers to a positive correlation; a ‘–’ refers to a negative correlation; a ‘0’ refers to no significant correlation.

Abbreviations: CC = Corpus Callosum; f = females; fr = frontal lobe; GM = gray matter; m = males; L = left; par = parietal lobe; R = right; read = reading; SGA: small for gestational age; spel = spelling; spl = splenium; temp = temporal lobe; WM = white matter.

### Oral language

#### White and gray matter volume

Five studies reported findings about total white matter (WM) and/or gray matter (GM) volumes and the correlation with oral language scores [20, 29–31, 33]. Four studies [29–31, 33] explicitly reported no significant correlations with WM volume in preterm children. However, one of these [29] did find a significant correlation for small for gestational age (SGA) children. Also, two studies [20, 31] found significant correlations in preterm children; the authors emphasized that these correlations are based on complicated, specific patterns of cortical and subcortical alterations. For example, Isaacs et al. described a positive correlation with WM volume in specific areas of the parietal and temporal lobes, a negative correlation between language and WM volume in frontal lobe areas, a negative correlation with GM volume in the parietal lobe and a positive correlation with GM volume in the frontal lobe [20]. Thus, both positive and negative correlations between oral language and GM and WM volume in different cortical areas were found.

#### Corpus callosum volume

Six studies described a relation between the volume of the corpus callosum (CC) and oral language skills. Four of them presented a positive correlation [24, 26, 27, 32]. Arhan et al. [24] even show a correlation of r = 0.91; p = 0.001, which can be interpreted as very strong. Caldu et al. [31] and Kontis et al. [36] did not find a significant correlation between oral language skills and CC volume.

#### Cerebellum volume

Four studies associated cerebellar volume with oral language skills. Arhan et al. [24] and Parker et al. [25] described a positive correlation between oral language skills and cerebellar volume in preterm children. Arhan et al. again show a very strong association (r = 0.93; p = 0.001). Two studies did not find a correlation in preterm children [29, 30]. Martinussen et al. [29] did find a correlation in SGA children though.

#### DTI measurements fasciculi

Five studies reported findings about the association between oral language skills and the fasciculi in the brain. None of the studies reported a significant relation between oral language skills and the arcuate fasciculus (AF). Moreover, two studies reported explicitly that no significant relation was found between oral language skills and the AF [32, 33]. However, three studies reported a significant positive relation between oral language and the uncinate fasciculus (UF) [32, 33, 35]. The correlations presented by Constable et al. [35] are worth to note specifically, since the associations they found were strong (for example r = -0.759; p = .003 for the association between PPVT scores and the right UF in females). One study reported a significant positive relation with the superior longitudinal fasciculus (SLF) [37].

### Verbal fluency: Language and executive functioning

#### White and gray matter volume

Five studies reported findings about the relation between WM and/or GM volume and verbal fluency skills. Two of these studies described a significant correlation [21, 22]. The one, by McCoy et al. [22], found a positive correlation in females, in higher temporal white matter. The other, by Nosarti et al. [21], found a positive correlation between WM volume in frontal and temporal regions and verbal fluency and a negative correlation between GM volume and verbal fluency. The other three studies did not find any correlation between verbal fluency and GM or WM volumes [23, 30, 31].

#### Corpus callosum volume

Five studies reported findings about the correlation between CC volume and verbal fluency. Three studies [26, 27, 36] described a positive correlation. However, Caldu et al. [31] did not find any correlation between CC and verbal fluency.

#### Cerebellum volume

None of the four studies that described the relation between cerebellar volume and verbal fluency found any correlation [22, 25, 28, 30].

#### DTI measurements fasciculi

Only Mullen et al. [33] reported about the relation between fasciculi and verbal fluency and found a significant positive correlation with the left and right AF and no correlation with the UF.

### Written language: Reading and spelling

#### White and gray matter volume

Four studies reported findings about GM and/or WM volume in relation with written language skills. Nosarti et al. [21] found significant correlations in the temporal gyrus: negative correlation with GM volumes and positive correlation with WM volumes in females only. Andrews et al. [34] also found a positive correlation in temporal parietal regions between reading and WM volume. Scott et al. [23] presented a positive correlation between GM volume in frontal lobe regions and no correlations, however, with WM volumes. Brumbaugh et al. [30] did not find significant correlations between WM volume and written language skills.

#### Corpus callosum volume

Andrews et al. [34] found a significant correlation between fractional anisotropy in the CC and reading skills. On the other hand, Nosarti et al. [27] did not find any significant correlations between CC volume and written language skills.

#### Cerebellum volume

Allin et al. [28] found a positive correlation between reading skills and cerebellar volume, but not between spelling skills and cerebellar volume. Brumbaugh et al. [30] did not find a correlation between reading and cerebellum volume.

#### DTI measurements fasciculi

Only one study, by Travis et al., described correlations between fractional anisotropy in the fasciculi and written language skills [15]. Correlations were found with reading and spelling and the left AF and left UF.

## Discussion

### Main findings

Our overview of study results in language and brain structure associations in preterm children yielded a complex set of relations, of which some show more consensus than others. We will discuss the most remarkable results.

Perhaps most notable is the lack of any association between structural brain injury and language outcomes. We had expected that preterm children with explicit brain damage would have the most severe language problems. However, in these studies brain damage was scored on a 3- or 4-point scale and naturally, in all studies the group of children with explicit damage was relatively small compared to groups of children with less damage, which makes it hard to prove a correlation with language. This might have influenced the correlations found between language skills and brain damage.

Another remarkable result concerns the cerebellum volume. We studied three language domains (i.e. oral language, verbal fluency and written language) and only very few studies found a significant correlation between any of these domains and the volume of the cerebellum; no correlation at all was reported for verbal fluency. A clear correlation between verbal fluency and cerebellar volume cannot be shown, and seems unlikely for both oral and written language.

The association of the CC volume with language outcomes is more convincing, particularly with regard to oral language skills and verbal fluency. Only one of the included studies did not find a significant correlation with language skills or verbal fluency [31], but this can likely be ascribed to insufficient statistical power, as this was the study with the smallest population of preterm children (N = 25). Overall, an association between oral language and CC volume is likely. However, the relation between CC and written language skills remain inconclusive, since there were only two studies that reported correlational data between these measures and they reported opposing results [27, 34].

Regarding the DTI studies, the most striking result is the repeatedly reported significant correlation between UF and oral language skills. The UF is part of the ventral language pathway and in recent literature it is often associated with semantic language functions. However, there is a lack of evidence for a general role of the UF in language [38]. Our review, though, showed a positive correlation between the UF and oral language skills. An association between language skills and the AF, which is part of the dorsal language stream, is less obvious according to our review results. Unfortunately, few studies included in our review addressed the role of the AF. Still, these tentatively show that the AF is more involved in verbal fluency, whilst the UF is more involved in oral language.

A less convincing result is the correlation between language and areas of WM and GM volume. Many studies did look at WM and GM in relation to one of the language domains, but the results were inconsistent. We propose that these kinds of differences between studies might arise because each addresses a slightly different microstructural area of the brain. When total WM or GM volume is studied, rarely any relation with language is found, while many significant relations are found when studying several microstructures of the brain. For example, Nosarti et al. [21] found a negative correlation between written language skills and GM volume in the temporal lobe, while Scott et al. [23] found a positive correlation in the frontal lobe. Overall we see that GM volume is more often negatively correlated with language skills, while WM volume mostly correlated positively with language skills. This negative association of GM with language corresponds with recent literature, also for example in stuttering literature [39], and has been associated with a cortical developmental phase of dendritic and synaptic pruning in late childhood and adolescence [40–43]. This might mark a shift from relatively diffuse cortical representations of cognitive functions in early childhood toward a more accurate, efficient, and faster processing language system later on.

Oral language skills are more often significantly correlated to preterm brain structures compared to verbal fluency skills or written language skills (see Table 2). Thus, atypical brain development in preterm children seems to affect oral language more obviously than it affects verbal fluency or reading or spelling. This is interesting in the sense that verbal fluency skills are also based on executive functioning, while oral language skills are mostly language proficiency tasks. Apparently, brain structures of preterm children are associated more strongly with language tasks than with executive functioning based language tasks.

### Influencing factors

It is plausible that gestational age (GA) is an important influencing factor in the relation between brain and language, where lower GA leads to more atypical brain and language development and a relation between these two parameters would be more obvious. However, the populations of almost all included studies consisted of very preterm children with a gestational age of <33 weeks. One study [30] included a population of late preterm children only (34–36 weeks GA). The authors did not find a correlation with language, which is in accordance with the idea that higher GA leads to less atypical brain and language development. However, this lack of correlation might be a consequence of the fact that only the cerebellum was studied, which in many of the other studies was not correlated with language. Because of these considerations we cannot indicate an effect of GA from our study results.

Another factor that might have been of influence is the MRI methodology used. Overall, studies that used DTI as a MRI measurement reported more significant correlations than studies that used volume measurements only.

A third factor to take into account is sex. Several included studies presented results for boys and girls separately. We analyzed these results to search for similarities in boys and girls, but did not find consensus within these results. Therefore, we cannot draw a general conclusion about the role of sex in the correlation between brain and language.

### Strengths and limitations

The strength of this review is that it provides a clear overview of the most comparable studies on the relation between language and brain structure in school-aged preterm children. This is a very complex subject because it covers two crossing parameters, which each in itself is complex and variable. We achieved to keep the most important factors relatively stable, such as age of the population, MRI scanner features, language tests used and population size. Also, our classification into three language domain groups resulted in a structured overview. We hope that this categorization will contribute to the validity of future correlational studies of brain function and language outcomes.

A possible limitation of the study is the risk of publication bias, i.e. studies may have analyzed more regions in the brain than reported in the result section. To partly adjust for this, when a certain region was mentioned in the methods section, but not addressed in the results section, we interpreted this as: no significant correlation found. And then, of course, performed studies that did not find any significant correlations may not have been published at all. Therefore, we highlighted the results presented by at least two study groups. When only one study looked into a certain relation–for example the relation between written language skills and the fasciculi—we did not highlight the outcome in our review results and discussion.

In our systematic review we chose to focus on the most commonly used MRI and DTI methods (structural T1- and T2-weighted sequences MRI and DTI). However, there are already some new models, such as non-tensor-based diffusion imaging analyses (e.g. high angular resolution diffusion imaging (HARDI)) that are very promising. HARDI is a new advanced model, which is an improvement with respect to DTI because it can deal with crossing fibers in voxels. It is successfully used very recently in a study with preterm children at term equivalent age [44]. The authors state that their findings suggest that differences in arcuate fasciculi micro-structure have a significant impact on language development and modulate the first stages of language learning. However, non-tensor-based diffusion imaging analyses were beyond the scope of our systematic review since the method is relatively new. Data are still limited and no studies are published in school-aged preterm children yet.

### Implications for further research

For future research we would recommend to relate the brain on a microstructural level to oral language skills in preterm children. We would recommend to use language tests such as the CELF, since this test battery consists of a number of subsets that cover all oral language domains and can be subdivided in subcategories, such as perceptive and expressive language. We recommend to study these oral language subcategories separately and relate these and written language skills to brain structures. With respect to MRI/DTI measurements, longitudinal GM and WM analysis seems to be promising methods, highly relevant to longitudinal research in preterm children and the relation with their cognitive development. We also recommend to use non-tensor-based diffusion imaging analysis, since this new advanced model is an improvement with respect to DTI. Besides, we recommend to use principle component analysis (PCA) as an analytical approach, which might prevent random correlational findings and can actually lead to meaningful associations. PCA is a renowned method with a longstanding tradition which is now again increasingly and successfully being used in neonatal MRI studies to quantify the proportion of shared variance in the measured water diffusion parameters (MD, FA, λ_ax_ and λ_rad_) across the tracts [45]. Lastly, more consistent data collection and data sharing could lead to more and quality-assured knowledge in this research field.

### Conclusion

This systematic review gives an overview of the extensive and diverse scientific literature on the associations between MRI brain measures and language outcome in children born preterm. Oral language skills and verbal fluency were shown to be associated with CC volume. Oral language skills are also associated with the UF. Overall, oral language skills are more obviously associated with several microstructural brain areas than are verbal fluency tasks, which are executive functioning based language tasks, and reading and spelling tasks. No associations were found between cerebellar volume and verbal fluency. The relation between oral language and written language with cerebellar development seems weak. The relation between preterm brain injury and language outcome could not be proven in studies that used brain damage scales. This most likely implies that not one single damaged brain area is responsible for atypical language development, but that several brain areas and their connections are essential. For future research we would recommend to study overall brain connectivity in combination with oral language skills, in which good quality management and data sharing will be crucial to enhance our shared knowledge and clinical opportunities.

## Supporting information

S1 Text Search protocol. (DOCX)

S1 Table Newcastle-Ottawa Quality Assessment Scale. (DOCX)

S2 Table PRISMA 2009 checklist. (DOC)
