# Correction: Language outcome related to brain structures in school-aged preterm children: A systematic review

**DOI:** 10.1371/journal.pone.0203298

**Published:** 2018-08-27

**Authors:** Lottie W. Stipdonk, Marie-Christine J. P. Franken, Jeroen Dudink

There is an error in Table 2. The column headings, “WM volume,” “GM volume,” “CC volume,” “Cerebellum volume,” and “DTI measurements Fasciculi” are missing. Additionally the row headings, “Oral language,” “Verbal Fluency (executive functioning and language),” and “Written language: (reading/spelling)” are missing.

**Table 2:** Study results. A ‘+’ refers to a positive correlation; a ‘–’ refers to a negative correlation; a ‘0’ refers to no significant correlation. Abbreviations: CC = Corpus Callosum; f = females; fr = frontal lobe; GM = gray matter; m = males; L = left; par = parietal lobe; R = right; read = reading; SGA: small for gestational age; spel = spelling; spl = splenium; temp = temporal lobe; WM = white matter.

	WM volume	GM volume	CC volume	Cerebellum volume	DTI measurements Fasciculi		
					Arcuate	Uncinate	Superior Longitudinal
**Oral language**	+SGA[29], +par/temp[20],–fr[20)	+[31], +fr[20]–par[20]	+spl[26], +[27]^,^ [24, 32]	+SGA[29], +[24, 25]		+[32, 33]L/R, +m[35],–f R[35]	+R[37]
	0[29, 31]^,^ [30]^,^[33]		0[31, 36]	0[29]^,^[30]	0[32, 33]L/R		
**Verbal Fluency (executive functioning and language)**	+fr/temp[21] +temp_f[22]	–fr/temp[21]	+[26, 27, 36]		+[33]L/R		
	0[30, 31], 0fr[23]	0[31]	0[31]	0[22, 25, 28, 30]		0[33]L/R	
**Written language: (reading/spelling)**	+fr/temp[21] +temp/par[34]	–fr/temp[21]+fr[23]	+[34]	+read[28]	+L[15]	+L[15]	+L/R[15]
	0[30], 0fr[23]		0[27]	0[30], 0spel[28]			
