# Supplementary material for: Language outcome related to brain structures in school-aged preterm children: A systematic review
Source: PLoS One. 2018 Jun 4;13(6):e0196607. doi: 10.1371/journal.pone.0196607 (PMC5986152; doi:10.1371/journal.pone.0196607)
Supplement: S1 Text — (DOCX) [file pone.0196607.s001.docx]

**Search protocol for each database, until January 2017**

| *Database* | *N articles* | *N unique articles* |
| --- | --- | --- |
| Embase.com | 1380 | 1351 |
| Medline Ovid | 944 | 194 |
| Web of science | 944 | 390 |
| Cochrane central | 50 | 0 |
| Google scholar | 200 | 148 |
| **Total** | **3518** | **2083** |

**Embase.com 1380**

('neurology'/exp OR brain/exp OR 'nuclear magnetic resonance imaging'/exp OR 'neurologic examination'/de OR neurophysiology/de OR (neurolog* OR neurophys* OR neurobiolog* OR (neuro NEXT/1 (phys* OR biolog*)) OR brain OR forebrain OR (cerebral* NOT cerebral-palsy) OR cerebell* OR 'corpus callosum' OR ((white OR grey OR gray ) NEXT/1 matter*) OR (magnetic* NEAR/3 resonan*) OR mri OR fmri):ab,ti) AND ('speech and language'/exp OR 'verbal behavior'/exp OR 'language disability'/de OR 'language ability'/de OR dyslexia/de OR 'developmental language disorder'/exp OR 'Peabody picture vocabulary test'/de OR 'speech disorder'/de OR 'academic achievement'/de OR 'language test'/exp OR (speech OR language* OR (verbal* NOT non-verbal*) OR linguist* OR reading OR writing OR literac* OR illiterac* OR vocabular* OR morphosynt* OR grammatic* OR phonolog* OR dysle* OR speak OR semantic* OR ((academic OR school) NEAR/3 achieve*)):ab,ti) AND ('immature and premature labor'/exp OR 'low birth weight'/exp OR 'intrauterine growth retardation'/de OR (((prematur* OR 'pre mature' OR 'pre maturity' OR preterm* OR 'pre term') NEAR/6 (infant* OR neonat* OR birth* OR child* OR newborn* OR baby OR babies OR born)) OR (low* NEAR/3 ('birth weight' OR birthweight)) OR LBW OR VLBW OR ELBW OR SGA OR (small NEAR/3 (age OR date)) OR IUGR OR ((intrauterine OR 'intra uterine') NEAR/3 (retard* OR restrict*))):ab,ti)

**Medline Ovid 944**

(exp "neurology"/ OR exp brain/ OR exp "Magnetic Resonance Imaging"/ OR exp "Magnetic Resonance Spectroscopy"/ OR "Neurologic Examination"/ OR Neurophysiology/ OR (neurolog* OR neurophys* OR neurobiolog* OR (neuro ADJ (phys* OR biolog*)) OR brain OR forebrain OR (cerebral* NOT cerebral-palsy) OR cerebell* OR "corpus callosum" OR ((white OR grey OR gray ) ADJ matter*) OR (magnetic* ADJ3 resonan*) OR mri OR fmri).ab,ti.) AND ("Speech-Language Pathology"/ OR "Language Disorders"/ OR "Language Development Disorders"/ OR exp "Language"/ OR exp "Verbal Behavior"/ OR "Language Tests"/ OR "Speech Disorders"/ OR (speech OR language* OR (verbal* NOT non-verbal*) OR linguist* OR reading OR writing OR literac* OR illiterac* OR vocabular* OR morphosynt* OR grammatic* OR phonolog* OR dysle* OR speak OR semantic* OR ((academic OR school) ADJ3 achieve*)).ab,ti.) AND (exp "Obstetric Labor, Premature"/ OR exp "Infant, Premature"/ OR exp "Infant, Low Birth Weight"/ OR "Fetal Growth Retardation"/ OR (((prematur* OR "pre mature" OR "pre maturity" OR preterm* OR "pre term") ADJ6 (infant* OR neonat* OR birth* OR child* OR newborn* OR baby OR babies OR born)) OR (low* ADJ3 ("birth weight" OR birthweight)) OR LBW OR VLBW OR ELBW OR SGA OR (small ADJ3 (age OR date)) OR IUGR OR ((intrauterine OR "intra uterine") ADJ3 (retard* OR restrict*))).ab,ti.)

**Cochrane central 50**

((neurolog* OR neurophys* OR neurobiolog* OR (neuro NEXT/1 (phys* OR biolog*)) OR brain OR forebrain OR (cerebral* NOT cerebral-palsy) OR cerebell* OR 'corpus callosum' OR ((white OR grey OR gray ) NEXT/1 matter*) OR (magnetic* NEAR/3 resonan*) OR mri OR fmri):ab,ti) AND ((speech OR language* OR (verbal* NOT non-verbal*) OR linguist* OR reading OR writing OR literac* OR illiterac* OR vocabular* OR morphosynt* OR grammatic* OR phonolog* OR dysle* OR speak OR semantic* OR ((academic OR school) NEAR/3 achieve*)):ab,ti) AND ((((prematur* OR 'pre mature' OR 'pre maturity' OR preterm* OR 'pre term') NEAR/6 (infant* OR neonat* OR birth* OR child* OR newborn* OR baby OR babies OR born)) OR (low* NEAR/3 ('birth weight' OR birthweight)) OR LBW OR VLBW OR ELBW OR SGA OR (small NEAR/3 (age OR date)) OR IUGR OR ((intrauterine OR 'intra uterine') NEAR/3 (retard* OR restrict*))):ab,ti)

**Web of science 944**

TS=(((neurolog* OR neurophys* OR neurobiolog* OR (neuro NEAR/1 (phys* OR biolog*)) OR brain OR forebrain OR (cerebral* NOT cerebral-palsy) OR cerebell* OR "corpus callosum" OR ((white OR grey OR gray ) NEAR/1 matter*) OR (magnetic* NEAR/2 resonan*) OR mri OR fmri)) AND ((speech OR language* OR (verbal* NOT non-verbal*) OR linguist* OR reading OR writing OR literac* OR illiterac* OR vocabular* OR morphosynt* OR grammatic* OR phonolog* OR dysle* OR speak OR semantic* OR ((academic OR school) NEAR/2 achieve*))) AND ((((prematur* OR "pre mature" OR "pre maturity" OR preterm* OR "pre term") NEAR/5 (infant* OR neonat* OR birth* OR child* OR newborn* OR baby OR babies OR born)) OR (low* NEAR/2 ("birth weight" OR birthweight)) OR LBW OR VLBW OR ELBW OR SGA OR (small NEAR/2 (age OR date)) OR IUGR OR ((intrauterine OR "intra uterine") NEAR/2 (retard* OR restrict*)))) )

**Google scholar**

neurological|neurophysology|neurobiology|forebrain|brain speech|language|verbal|linguistic|reading|writing|literacy|illiteracy|vocabulary|grammatics|phonology|dyslexia premature|prematurity|preterm|"low birth|birthweight"|"small*gestational age"
