# Supplementary material for: Language outcome related to brain structures in school-aged preterm children: A systematic review
Source: PLoS One. 2018 Jun 4;13(6):e0196607. doi: 10.1371/journal.pone.0196607 (PMC5986152; doi:10.1371/journal.pone.0196607)
Supplement: S1 Table — (DOCX) [file pone.0196607.s002.docx]

|  | **Selection** | | | | | **Comparability** | | **Outcome** | | | **Total score** |
| --- | --- | --- | --- | --- | --- | --- | --- | --- | --- | --- | --- |
|  | Representativeness of exposed cohort | Selection of the non-exposed cohort | Ascertainment of exposure | Outcome of Interest not present at start study? | Controls matched on age? | | Control matched on other risk factors | Assessment of outcome | Follow-up long enough for outcome to occur? | Adequacy of follow-up of cohort? |  |
| *Acceptable (*)* | *Representative study group for very preterm born children* | *Control group drawn from same birth cohort* | *MRI/DTI data and language test* | *Correct* | *Yes* | | *Yes* | *Independent or blind assessment stated in the paper* | *From schoolage (6-17 years)* | *Complete follow-up, or subjects lost to follow-up unlikely to cause bias* |  |
| Gäddlin et al. | * | * | * | * | * | | - | - | * | * | 7 |
| Yliherva et al. | * | * | * | * | * | | * | * | * | - | 8 |
| Rushe et al. | * | * | * | * | * | | - | - | * | * | 7 |
| Skranes et al. | * | - | * | * | - | | - | - | * | * | 5 |
| Isaacs et al. | * | - | * | * | - | | - | - | * | * | 5 |
| Nosarti et al. (2008) | * | * | * | * | * | | * | - | * | * | 8 |
| McCoy et al. | * | - | * | * | - | | - | * | * | * | 6 |
| Scott et al. | * | * | * | * | * | | - | - | * | * | 7 |
| Arhan et al. | * | - | * | * | * | | * | * | * | * | 8 |
| Parker et al. | * | - | * | * | * | | - | - | * | * | 6 |
| Narberhaus et al. | * | - | * | * | * | | * | - | * | - | 6 |
| Noasrti et al. (2004) | * | - | * | * | * | | - | - | * | * | 6 |
| Allin et al. | * | - | * | * | * | | - | - | * | * | 6 |
| Martinussen et al. | * | - | * | * | * | | - | - | * | * | 6 |
| Brumbaugh et al. | * | * | * | * | * | | * | - | * | * | 8 |
| Caldu et al. | * | - | * | * | * | | * | - | * | * | 7 |
| Northam et al. | * | - | * | * | * | | * | - | * | - | 6 |
| Mullen et al. | * | - | * | * | * | | * | * | * | * | 8 |
| Andrews et al. | * | - | * | * | * | | - | - | * | * | 6 |
| Constable et al. | * | - | * | * | * | | * | - | * | * | 7 |
| Kontis et al. | * | - | * | * | * | | - | - | * | - | 5 |
| Skranes et al. | * | * | * | * | * | | - | * | * | * | 8 |
| Travis et al. | * | * | * | * | * | | * | - | * | - | 7 |

Newcastle-Ottawa Quality Assessment Scale
